# Efficacy of triple dose albendazole treatment for soil-transmitted helminth infections

**DOI:** 10.1371/journal.pone.0272821

**Published:** 2022-08-12

**Authors:** Mian Zi Tee, Soo Ching Lee, Yi Xian Er, Nan Jiun Yap, Romano Ngui, Alice V. Easton, Vinnie Wei Yin Siow, Kee Seong Ng, Christopher Chiong Meng Boey, Kek Heng Chua, Ken Cadwell, P’ng Loke, Yvonne Ai Lian Lim

**Affiliations:** 1 Department of Biomedical Science, Faculty of Medicine, University of Malaya, Kuala Lumpur, Malaysia; 2 Type 2 Immunity Section, Laboratory of Parasitic Diseases, National Institute of Allergy and Infectious Diseases, National Institutes of Health, Bethesda, MD, United States of America; 3 Department of Parasitology, Faculty of Medicine, University of Malaya, Kuala Lumpur, Malaysia; 4 Department of Microbiology, New York University Grossman School of Medicine, New York, NY, United States of America; 5 Department of Gastroenterology, Faculty of Medicine, University of Malaya, Kuala Lumpur, Malaysia; 6 Department of Paediatrics, Faculty of Medicine, University of Malaya, Kuala Lumpur, Malaysia; 7 Kimmel Center for Biology and Medicine at the Skirball Institute, New York University Grossman School of Medicine, New York, NY, United States of America; 8 Division of Gastroenterology, Department of Medicine, New York University Langone Health, New York, NY, United States of America; Universidade Federal de Minas Gerais, BRAZIL

## Abstract

In Malaysia, soil-transmitted helminth (STH) infections still persist among indigenous communities. In the past, local studies have focused mostly on epidemiologic aspects of STH infections with a scarcity of information on the efficacy of deworming treatment. The present study consisted of 2 phases: a cross-sectional phase on current epidemiological status and risk factors of STH infections and a longitudinal study over 6 weeks on triple dose albendazole efficacy against STH infections. A total of 253 participants were recruited at baseline and a pre-tested questionnaire was administered to obtain information on socio-demographics, environmental and behavioural risk factors. Stool samples were evaluated using a modified Kato-Katz technique. Cure rate (CR) and egg reduction rate (ERR) were assessed at 3 weeks following a 3-day course of 400mg albendazole treatment and infection status were observed again at 6 weeks. Baseline positivity of trichuriasis, ascariasis and hookworm infections were 56.1%, 11.9% and 20.2%, respectively. Multivariate analysis showed age below 18 years old (*P* = 0.004), without latrine in house (*P* = 0.042) and indiscriminate defecation (*P* = 0.032) were associated with STH infections. In the longitudinal study (N = 89), CR for trichuriasis was 64.6%, while CR of 100% was observed for both ascariasis and hookworm. ERR was above 90% for all three STH species. A rapid increased of *Trichuris trichiura* egg output was observed at 6 weeks. In conclusion, STH infections are highly prevalent among indigenous communities. Children and teenagers, poor sanitation and hygiene behaviour were determinants for STH infections. Triple dose albendazole is found to be efficacious against *Ascaris lumbricoides* and hookworm infections but has moderate curative effect with high ERR against *T*. *trichiura*. Although triple dose albendazole regimen has logistic challenges and may not be a routine option, consideration of this treatment regime may still be necessary in selective communities to reduce high intensity of *T*. *trichiura* infection.

## Introduction

Soil-transmitted helminth (STH) infections are classified by the World Health Organization (WHO) as neglected tropical diseases, affecting approximately 24% of the world’s population. The four main species that infect humans are *Ascaris lumbricoides*, *Trichuris trichiura* and hookworms (*Ancylostoma duodenale* and *Necator americanus*) [1]. In the Southeast Asian (SEA) countries, a high number of soil-transmitted helminthiasis cases had been reported, with ascariasis being the most prevalent, followed by trichuriasis and hookworm infections, respectively [2, 3]. Infections are principally related to inadequate water facilities, poor sanitation and low standards of hygiene [4].

While socioeconomic and infrastructural advancements have witnessed reduction of STH in urban population in Malaysia [5], the foci of high endemicity of STH is still persistent in rural areas, especially among indigenous communities, known as Orang Asli [6, 7]. In the past decades, epidemiology of STH infections studied among indigenous communities have recorded prevalence rate of more than 50% [7–11]. Despite efforts made by the government to improve their living conditions and socioeconomic levels by initiating the Resettlement Plans Schemes (RPS), providing basic social amenities, education and healthcare facilities, the intended impact unfortunately has not been obvious [6, 7]. The persistence of high STH prevalence could be due to high environmental contamination and behavioural factors [12].

Generally, STH infections are not fatal but associated with high morbidity rates. Morbidity corresponds with helminth burden (intensity of infection) [12]. While light infections often remain asymptomatic, chronic infections (moderate-to-heavy intensity) can lead to malnourishment, impaired cognitive development, stunted growth and anaemia [13–16]. With the aim of curtailing the morbidity, the World Health Organisation (WHO) published new target for STH control in 2020, to achieve a prevalence of moderate-to-heavy infections less than 2% by 2030 [17]. Preventive chemotherapy (PC) is the hallmark of one of the STH control strategies endorsed by the WHO which is based on periodic distribution of large-scale anthelmintic drugs to populations at risk living in endemic areas [18].

In 1974, Malaysia launched the National Worm Control Programme after intestinal helminth infections were recognised as major public health concerns [19]. In this programme, single dose of pyrantel pamoate was given to more than 3 million of schoolchildren from rural areas, including Orang Asli communities [6, 19]. Nevertheless, the programme was discontinued in 1983 due to low efficacy of pyrantel pamoate against *T*. *trichiura* and hookworm and insufficient monitoring of the programme [6]. Generally, children in rural and aboriginal settlements which are endemic for these infections are still able to receive anthelmintic drug through community health campaign by the Ministry of Health. Anthelmintic treatment is also administered at maternal and child clinics or mobile clinic services as well as from their school health services upon request [8, 20]. With the high persistence of STH prevalence in aboriginal settlements, mass deworming programme should be re-instituted to control and eliminate the STH infections.

Currently, benzimidazole (albendazole or mebendazole) are used in virtually all PC programmes as a single dose regimen, due to its cost effectiveness, minor side effects and ease of administration by non-medical personnel [1]. Although single dose albendazole is highly efficacious against *A*. *lumbricoides* (CR and ERR >95.0%) and had a reasonably performance against hookworm (CR = 79.5%) [21], poor curative effect against *T*. *trichiura*. (CR 1.1–50.0%) has been reported in previous studies [21–26]. A remarkably low CR of 5.5% against *T*. *trichiura* was reported for single dose albendazole treatment among Orang Asli children in Malaysia [27]. This raises the concern of the efficacy of a single dose albendazole regimen. In recent years, a number of studies have reported an improvement of the drug efficacy in multiple dose regimens [23, 25, 28]. However, there is a paucity of literature available on multiple dose efficacies of albendazole against STH in Malaysia, particularly in Orang Asli communities where these infections are very prevalent. Although triple dose regimens of albendazole was attained in studies by Al-Mekhlafi et al. [29] and Ramanan et al. [30] in Orang Asli communities, the focus of these studies was on post treatment reinfection of STH while the latter investigated effect of helminth clearance on gut microbiota following albendazole treatment.

Within this context, we conducted a longitudinal epidemiological study to investigate the current status and risk factors of STH infections as well as the therapeutic efficacy of triple dose albendazole among Orang Asli communities in Malaysia. Post sampling was taken at 21 days (3 weeks) after drug administration in accordance to WHO published guideline (14–21 days) to avoid biased estimation of ERR from residual eggs released by degenerating worms [31, 32]. Besides, transient inhibition of albendazole against *T*. *trichiura* following treatment has been observed in previous studies [33, 34]. Hence, the efficacy of albendazole was further examined at 6 weeks. It is hoped that the findings of this study would be beneficial for public health officials to develop effective targeted and customised control programmes to reduce STH infection significantly in this highly endemic communities.

## Methods

### Ethics statements

This study was approved by both the Medical Ethics Committee of University of Malaya Medical Centre (UMMC) (Reference No.: 2017925–5593) and National Medical Research Register (NMRR), Ministry of Health, Malaysia (Reference No.: NMRR-17-3055-37252). Permission was obtained from the Department of Orang Asli Development (JAKOA) [Reference No.: JAKOA/pp.30.052Jld13 (12) & JAKOA/pp.30.052Jld14 (47)] and the chieftain of the respective villages (referred to as Tok Batin) prior to commencement of this study. There are no deviations from the study protocol after the approval was obtained. The purpose and procedure of this study were explained to the villagers before the study was initiated. Prior to inclusion in this study, written consent was obtained from all participants and as for their children under 18 years old, consent was attained from their respective parents or guardian. In addition, assent was obtained from children from 7–18 years old.

#### Inclusivity in global research

Additional information regarding the ethical, cultural, and scientific considerations specific to inclusivity in global research is included in the Supporting Information (S1 Checklist).

### Study area

This is a longitudinal study conducted between May 2018 and August 2019 at four different Orang Asli villages, one from Perak and three from Selangor states in Peninsular Malaysia. The villages include Rasau (Perak state), Sungai Judah, Tanjung Sepat and Bukit Bangkong. These four villages were selected from an official village list provided by JAKOA taking into consideration (i) the accessibility by road, (ii) population of more than 100 people and (iii) agreement of village chieftain. These criteria were taken into account as longitudinal study required frequent visits to the village and there could be a high dropout rate at subsequent follow-up time-points. These communities are from the Senoi tribe, comprising of Semai sub-tribe (Perak) and Mah Meri sub-tribe (Selangor). In general, all the study villages are considered as suburban areas, except village Rasau which is in a rural area. These villages are surrounded by oil palm or rubber plantations and Rasau is also located at the fringe of a forest. In general, all the villages are equipped with electricity and piped water supply. The houses in these villages are made of brick or traditional structures of bamboo or wooden house. In term of language, the villagers were still speaking in their native language, however, they have a good command of the national (Malay) language.

### Study design and samples collection

The sample size was calculated using the formula described in Charan & Biswas [35] based on the estimated prevalence at 81% taken from previous study [36], with 95% level of confidence and 5% margin of error. The minimum sample size required to enrol at baseline for cross sectional study was 236. To evaluate the efficacy of albendazole drugs, sample size was calculated based on WHO guideline [32]. A minimum sample of 50 positive for each helminth species is required for drug efficacy evaluation. The minimum number to be screened was estimated at 82, 240 and 160 for *T*. *trichiura*, hookworm and *A*. *lumbricoides*, respectively. The calculation was based on conservative compliance rate of 80% and prevalence of 76.6% for *T*. *trichiura*, 26.4% for hookworm and 39% for *A*. *lumbricoides* taken from previous studies [36, 37].

Study inclusion criteria included healthy individuals aged 4 to 85 years old, and resided in the village for at least 1 year. Exclusion criteria included pregnant women and breastfeeding mothers. This is a longitudinal follow up which consisted of 3 time-points: baseline, 3 weeks and 6 weeks after deworming treatment. A clean, wide-mouth screw-cap pre-labelled stool containers with name and identity code were distributed to the participants along with proper instruction on collecting stool samples. The stool samples were collected the next day. After stool samples were collected, all the participants were treated with a 3-day course of 400mg albendazole and were being observed by the researcher (direct observed therapy, DOT). Ten participants were not present for the third oral administration due to work commitments but were allowed to consume albendazole at home. Stool samples were collected at 3 weeks and 6 weeks post-treatment from the same participants. All samples collected were kept in a cooler with ice packs while being transported back to the University of Malaya and processed on the day of collection. In a more remote village in Perak state, samples were stored at 4°C for 2 days before being transferred back to the laboratory for immediate processing. This could have underestimate STH infections, especially hookworm.

### Pre-tested questionnaire

A dual language pre-tested questionnaire in English and Malay was administered face-to-face in Malay language once at baseline. For young children, the questionnaire was administered with the help of their parents or guardians. Information collected included demographic data, socioeconomic status, educational level, source of water supply, sanitation and environmental condition, personal hygiene practice, owned/close contact with domestic animals and history of medication.

### Helminth infection status

A modified Kato-Katz technique was performed as reported in [38] to determine the helminth infection status of participants. A thick smear was prepared from the fresh stool according to manufacturer’s instructions of the Kato-Katz kit, Mahidol University, Thailand. In brief, stool sample was pressed through a stainless sieve, with a sieve opening of 420μm. The sieved stool was transferred to a template which deliver 39.2mg of stool [38] on the glass slide. The stool sample was covered with glycerin-malachite green-soaked cellophane and pressed to spread the stool evenly across the surface. Single slide was prepared and egg counts were cross checked by a second personnel. The slide was examined under a microscope within 30–60 minutes after preparation for hookworm eggs to prevent clearing of hookworm eggs while *T*. *trichiura* and *A*. *lumbricoides* eggs were examined after 60 minutes. The number of helminth eggs were counted separately for *T*. *trichiura*, *A*. *lumbricoides* and hookworm. As the template delivered 39.2mg of stool, total number of egg counts were multiplied by 25.5 to generate eggs per gram (EPG) of stool. The parasitological status of participants at each time-point was calculated and expressed in terms of infection intensity [arithmetic mean (AM) eggs per gram of stool (EPG)]. Standard error was computed using descriptive statistical analysis in SPSS. Infection intensity was stratified into light, moderate or heavy according to WHO cut-offs [39].

### Statistical analysis

Statistical analysis was performed by using Statistical Package for the Social Sciences (SPSS) software programme for Windows version 22 (SPSS Inc., Chicago, IL, USA). The display graphs were performed using R Statistical Software (version 4.0.3) along with R Studio Software (version 1.3.1093). For descriptive analysis, general characteristics of the study population (demographic, socio-economic, environmental and personal hygiene practice) and positivity rate of infections were expressed as a percentage. Normality of all continuous variables was tested by Shapiro-Wilk along with QQ-plot or histogram. The normality was set as P>0.05. The normal distributed data were presented as mean (standard deviation; SD) while not normally distributed data were presented as median (interquartile range; IQR). The confidence interval (CI) of proportion was calculated based on Clopper-Pearson method.

In univariate analysis, Person’s chi-square test was computed on dichotomous scale to investigate the risk association between STH infection (dependent variable) and independent variables which include socio-demographic factors, environmental factors, personal hygiene practices and owning pets/domestic animal [40]. Variables with significant level (P<0.05) were further included in the multivariate analysis using binomial logistic regression and measured on dichotomous scale. In addition, variables with borderline significant level of P<0.25 were also included in multivariate analysis to avoid potential risk factors being excluded and also due to the low number of variables with P<0.05. The model fitness was determined by Hosmer-Lemeshow statistic [7]. Data were interpreted using adjusted OR and 95% CI. The level of significance value was set as P<0.05.

The efficacy of albendazole treatment was determined by CR and ERR at 3 weeks post-treatment by using the formula as described in Zeleke et al. [41]. The CR was defined as number of individuals positive at baseline and become negative after treatment. ERR was referred to relative reduction of mean eggs output after treatment compared to the mean eggs output at baseline. The effects of albendazole on STH positivity and egg output was also assessed at 6 weeks after treatment. Since egg production for *T*. *trichiura* and *A*. *lumbricoides* take 2–3 months after a new infection and 5–8 weeks for hookworm infection [42–44], we are not technically measuring reinfection, which is defined as recurrence caused by a different organism that was initially present [45]. Additionally, limitations to the sensitivity of the Kato-Katz smear test may falsely indicate reinfection, if initial post treatment samples were false negatives. In order to address these issues, if an egg-negative individual has an increased egg count at the 6 weeks time point, we defined him/her as an egg-positive individual instead of categorizing the individual as reinfected individuals. Subjects who were initially negative and became positive after treatment was denoted as new positive individuals.

## Results

### Participant flow

A total of 253 participants were recruited at baseline. Participants were treated with 400mg albendazole for 3 consecutive days. At 3 weeks post treatment, 148 participants were excluded because they failed to provide stool sample or failed to complete the three doses while 105 participants who provide stool samples and fully treated with 3 doses of albendazole were enrolled in the study. At 6 weeks, 16 participants were excluded from the study for not providing stool sample. Hence, a total number of 89 participants were included in the final analysis.

### General demographic profiles, socio-economic, environmental and sanitary behaviour characteristics of the population at first time point (baseline)

The general demographic profile and characteristics of the participants are shown in Table 1 (further information is available in S1 Appendix). A total of 253 participants were recruited in this study but only 54.2% of participants completed all the questions in the questionnaire (70% completed 80% of the questionnaire). Despite having higher percentage of female (53.4%) who did not complete the questionnaire in comparison to male (46.6%), there is no significant association between gender and completion of questionnaire (P = 0.167). Of the 253 participants, 58.1% were female and 41.9% were male. The participants ranged from approximately 4 to 81 years of age, with a median age of 32 years (IQR 37 years). About half of them (52.6%) were not working, where most were students and housewives. Majority of the participants (83.4%) belonged to households with monthly household income of less than RM2,208, which is below the poverty income threshold in Malaysia [43]. With regards to educational attainment, 62.9% had formal education, however most of them did not continue or complete secondary school. In general, about 70% of the participants lived in houses equipped with basic amenities, such as piped water supply and 64% had household toilet. However, a small number of them were dependent on the river as a water source (7.5%) and practiced open defecation (11.5%). Besides, more than half of the participants (60.5%) practiced open burning as a means of garbage disposal. Of 150 participants who owned domestic animals, 82% of them allowed the animals to defecate indiscriminately. Detailed demographic date and socio-economic characteristic of the population were presented in Table 1.

**Table 1:** Demographic profiles, socio-economic, environmental and sanitary behaviour characteristics of study populations.

Variables		n (%)	95% CI
General Demographic			
Village	Rasau	48 (19.0)	14.3–24.4
	Sungai Judah	116 (45.9)	39.6–52.2
	Tanjung Sepat	42 (16.6)	12.2–21.8
	Bukit Bangkong	47 (18.6)	14.0–23.9
Gender	Male	106 (41.9)	35.8–48.2
	Female	147 (58.1)	51.8–64.3
Age groups	< 6 (young children)	22 (8.7)	5.5–12.9
	7–12 (school aged children)	51 (20.2)	15.4–25.6
	13–17 (teenagers)	9 (3.6)	1.6–6.6
	> 18 (adults)	168 (66.4)	60.2–72.2
	Not answered	3 (1.2)	0.3–3.4
Socio-economic & Education			
Occupational status	Not working^a^	133 (52.6)	46.2–58.9
	Non-skilled worker	25 (9.9)	6.5–14.2
	Farm worker/Rubber plantation	20 (7.9)	4.9–11.9
	Fisherman	16 (6.3)	3.7–10.1
	Others^b^	11 (4.4)	2.2–7.7
	Not answered	48 (19.0)	14.3–24.4
Monthly household income (RM)	Less than RM2208	211 (83.4)	78.2–87.8
	More than RM2208	8 (3.2)	1.4–6.1
	Not answered	34 (13.4)	9.5–18.3
Educational level	No formal education	46 (18.2)	13.6–23.5
	Pre-school	9 (3.6)	1.6–6.7
	Primary education	101 (39.9)	33.8–46.2
	Secondary education	48 (19.0)	14.3–24.4
	Tertiary education	1 (0.4)	0.0–2.2
	Not answered	48 (19.0)	14.3–24.4
Environmental and sanitary behaviour			
Source of water supply	Treated water	179 (70.8)	64.7–76.3
	Untreated water^c^	19 (7.5)	4.6–11.5
	Not answered	55 (21.7)	16.8–27.3
Latrine facilities	Yes	162 (64.0)	57.8–70.0
	No	36 (14.2)	10.2–19.2
	Not answered	55 (21.7)	16.8–27.3
Defecation sites	Latrine	174 (68.8)	62.7–74.4
	Open/ indiscriminate^d^	29 (11.5)	7.8–16.1
	Not answered	50 (19.8)	15.0–25.2
Garbage disposal	Proper	50 (19.8)	15.0–25.2
	Indiscriminate^e^	153 (60.5)	54.2–66.5
	Not answered	50 (19.8)	15.0–25.2
Own pets/domestic animals			
Own pets/domestic animals	Yes	150 (59.3)	53.0–65.4
	No	77 (30.4)	24.8–36.5
	Not answered	26 (10.3)	6.8–14.7
Defecation sites of domestic animals	Proper place	27 (10.7)	7.2–15.2
	Indiscriminate	123 (48.6)	42.3–55.0
	No pets	77 (30.4)	24.8–36.5
	Not answered	26 (10.3)	6.8–14.7
Close contact with domestic animals	No	65 (25.7)	20.4–31.5
	Yes	65 (25.7)	20.4–31.5
	No pets	77 (30.4)	24.8–36.5
	Not answered	46 (18.2)	13.6–23.5

^a^ Mainly housewives, students, children, retirees and unemployed individuals

^b^ Skilled workers and government servants

^c^ Untreated water include river and rainwater

^d^ Open defecation, commonly near the stream and bushes

^e^ Open burning

CI = Confidence interval

### Positivity and intensity of STH infections

Overall, 62.1% (95% CI = 55.8–68.1%) of 253 participants were infected with at least one STH species (Table 2). The most dominant STH species was *T*. *trichiura* (56.1%; 95% CI = 49.8–62.3%), followed by hookworm (20.2%; 95% CI = 15.4–25.6%) and *A*. *lumbricoides* (11.9%; 95% CI = 8.2–16.5%) (Table 2; S1 Appendix). As for polyparasitism status, coinfection of *T*. *trichiura* and hookworm were the most common in double infection, accounting for 10.7% (95% CI = 7.2–15.2). In addition, 5.1% (95% CI = 2.8–8.6) of the participants had triple infection. With regards to intensity, most of the participants who had STH infections had light infection burden (69.0%; 95% CI = 60.7–76.5% trichuriasis; 60.0%; 95% CI = 40.6–77.3% ascariasis; 86.3%; 95% CI = 73.7–94.3% hookworm infections) (Table 3). In general, both trichuriasis and ascariasis had similar pattern of worm burden, with about one third and less than 10% had moderate and heavy infection, respectively. On the other hand, 11.8% and 2.0% of the infections by hookworm were of heavy and moderate intensity, respectively. According to STH positivity rate at individual villages, high positivity rate of more than 50% was demonstrated in three surveyed villages (i.e., Rasau, Sungai Judah and Tanjung Sepat), whereas Bukit Bangkong had the least (14.9%) (Table 4). With regards to specific age groups, young children were highly infected with STH infections (81.8%; 95% CI = 59.7–94.8%) while the adult group had the lowest infections (56.0%; 95% CI = 48.1–63.6%).

**Table 2:** Overall STH positivity among Orang Asli community (N = 253).

STH species	Total (N = 253)	
** **	**n (%)**	**95% CI**
Overall STH infection	157 (62.1)	55.8–68.1
**STH infection (by species)**		
*T*. *trichiura*	142 (56.1)	49.8–62.3
*A*. *lumbricoides*	30 (11.9)	8.2–16.5
Hookworm	51 (20.2)	15.4–25.6
**Mono & Polyparasitism status (by species)**		
*T*. *trichiura*	91 (36.0)	30.1–42.2
*A*. *lumbricoides*	4 (1.6)	0.4–4.0
Hookworm	9 (3.6)	1.6–6.7
*T*. *trichiura + A*. *lumbricoides*	11 (4.3)	2.2–7.7
*T*. *trichiura* + Hookworm	27 (10.7)	7.2–15.2
*A*. *lumbricoides* + Hookworm	2 (0.8)	0.1–2.8
*T*. *trichiura* + *A*. *lumbricoides* + Hookworm	13 (5.1)	2.8–8.6

CI = Confidence interval

**Table 3:** Intensity of STH infections of the study population at baseline (N = 253).

STH species		Baseline (N = 253)		
		n (%)	95% CI	Arithmetic mean EPG (SE)
Overall STH infection		157 (62.1)	55.8–68.1	NA
*T*. *trichiura*				
Overall		142 (56.1)	49.8–62.3	4,206.42 (1,285.50)
Light		98 (69.0)	60.7–76.5	328.77 (26.42)
Moderate		33 (23.2)	16.6–31.1	2,751.68 (333.05)
Heavy		11 (7.7)	3.9–13.4	43,117.02 (11,585.71)
*A*. *lumbricoides*				
Overall		30 (11.9)	8.2–16.5	13,649.73 (3,954.93)
Light		18 (60.0)	40.6–77.3	130.33 (74.13)
Moderate		10 (33.3)	17.3–52.8	25,692.53 (3,684.45)
Heavy		2 (6.7)	0.8–22.1	75,110.25 (6,693.75)
Hookworm				
Overall		51 (20.2)	15.4–25.6	1,639.75 (519.50)
Light		44 (86.3)	73.7–94.3	331.50 (44.69)
Moderate		1 (2.0)	0.1–10.5	NA
Heavy		6 (11.8)	4.4–23.9	10,950.13 (1,694.30)

EPG = Eggs per gram; SE = Standard error; NA = Not applicable

**Table 4:** STH positivity according to village and age groups.

Variables	N	n (%)	95% CI
Overall STH infection	253	157 (62.1)	55.8–68.1
**Village**			
Rasau	48	43 (89.6)	77.3–96.5
Sungai Judah	116	83 (71.6)	62.4–79.5
Tanjung Sepat	42	24 (57.1)	41.0–72.3
Bukit Bangkong	47	7 (14.9)	6.2–28.3
**Age**			
< 6 (young children)	22	18 (81.8)	59.7–94.8
7–12 (school aged children)	51	36 (70.6)	56.2–82.5
13–17 (teenagers)	9	6 (66.7)	29.9–92.5
> 18 (adults)	168	94 (56)	48.1–63.6
NA	3	NA	NA

CI = Confidence interval; NA = Not applicable

### Risk factors of STH infections

**Fig 1:**
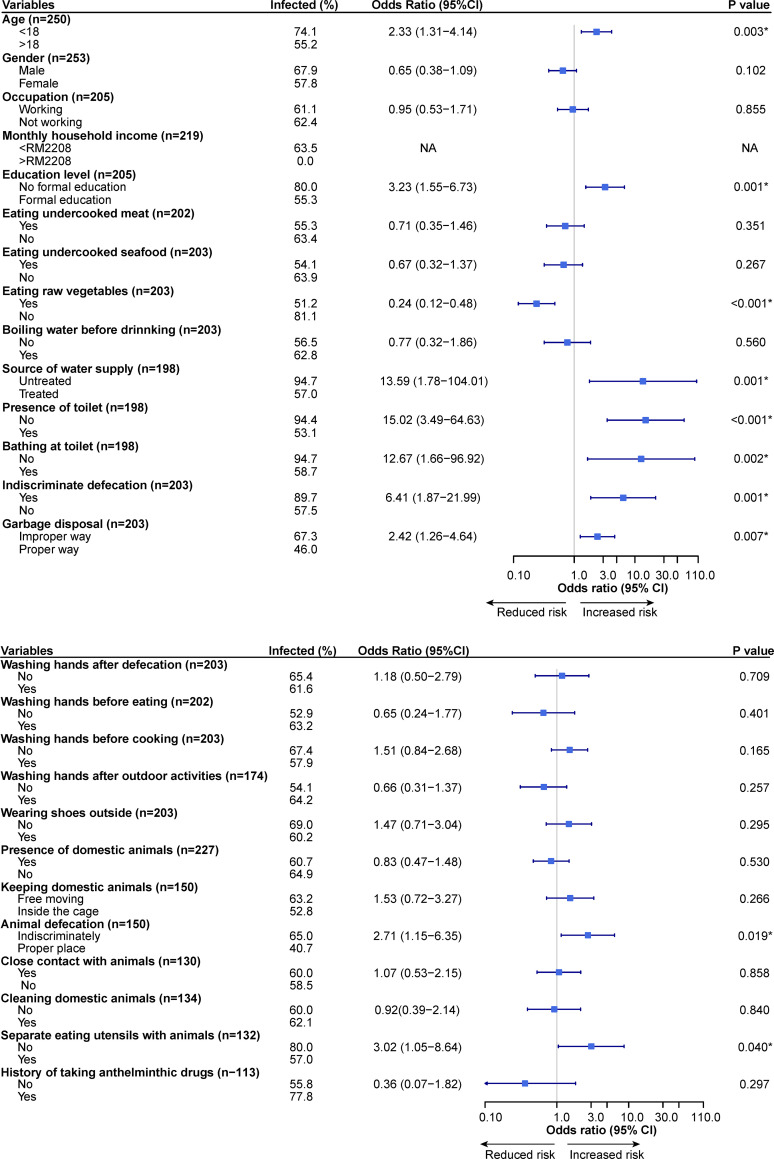
Forest plot of univariate analysis of potential risk factors associated with STH infections among the study population. CI = Confidence interval; * = Significant risk factors (*P*<0.05).

Results of univariate analysis for the association of STH infections with demographic, socio-economic, environmental and personal hygiene factors are shown in Fig 1. The results showed that participants who were below 18 years old showed significant association with STH infections (*P* = 0.009). In term of socio-economic factors, no formal education (*P* = 0.001) was found associated with STH infections. Other significant risk factors were related to the lack of basic facilities and poor hygiene practice, including the usage of untreated water supply (*P* = 0.001), lack of latrine in the house (*P*<0.001), not taking shower in bathroom (*P* = 0.002), defecating indiscriminately (*P* = 0.001) and improper garbage disposal (*P* = 0.007). Surprisingly, a diet lacking raw vegetables was associated with STH infections (*P*<0.001). With regards to some participants who owned pets, allowing their pets to defecate indiscriminately (*P* = 0.019) and sharing the eating utensils with their pets (*P* = 0.040) were two of the risk factors associated with STH infections.

**Fig 2:**
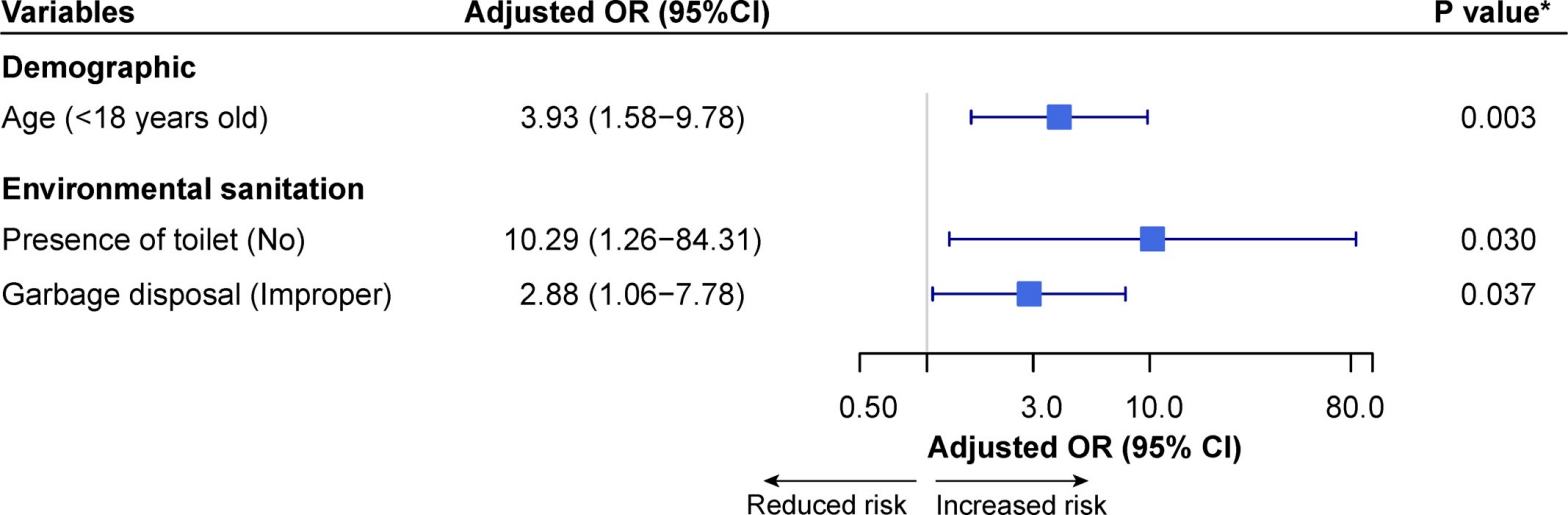
Forest plot of multivariate analysis of potential risk factor associated with STH infections among the study population. Adjusted OR = Adjusted Odds ratio; CI = Confidence interval; * = Significant risk factors (*P*<0.05).

Multiple logistic regression findings are shown in Fig 2. From the variables with p<0.25 shown in univariate analysis, three factors were retained as significant predictors in multivariate model, confirming that age below 18 years old (OR = 3.79, *P* = 0.004), lack of latrine in the house (OR = 9.11, *P* = 0.042) and indiscriminate defecation (OR = 5.73, *P* = 0.032) were significant risk factors associated with STH infections.

### Treatment efficacy of triple dose albendazole against STH infections

To evaluate the performance of triple dose albendazole against STH, only participants who provided stool samples for 3 time-points (baseline, 3 weeks and 6 weeks post-treatment) and fully treated with 3 doses of albendazole were included. In total, 89 participants completed the 3 time-points. The number of participants with different infection intensities as well as the arithmetic mean of EPG and changes following treatments are shown in Table 5 (for details, see dataset in S2 Appendix).

**Table 5:** Positivity rates, infection intensities, cure rates and egg reduction rates of *T*. *trichiura*, *A*. *lumbricoides* and hookworm at baseline, 3 weeks post-treatment and 6 weeks post-treatment among who were fully treated with 3 doses of albendazole (N = 89).

	*T*. *trichiura*			*A*. *lumbricoides*			Hookworm		
	n	% (95% CI)	EPG range^¥^	n	% (95% CI)	EPG rangeφ	N	% (95% CI)	EPG rangeᶲ
Baseline									
Infection intensity									
Light	32	35.9 (26.1–46.8)	25.5–969.0	7	7.9 (3.2–15.5)	25.5–76.5	9	10.1 (4.7–18.3)	25.5–918.0
Moderate	13	14.6 (8.0–23.7)	1,032.8–5,100.0	4	4.5 (1.2–11.1)	27,591.0–48,934.5	1	1.1 (0.0–6.1)	3,340.5
Heavy	3	3.4 (0.7–9.5)	19,099.5–33,099.0	1	1.1 (0.0–6.1)	81,804.00	3	3.4 (0.7–9.5)	7,650.0–18,181.5
Total positivity	48	53.9 (43.0–64.6)	25.5–33,099.0	12	13.5 (7.2–22.4)	25.5–81,804.0	13	14.6 (8.0–23.7)	25.5–18,181.5
Arithmetic mean EPG	2,427.5			18,812.6			3,005.1		
3 weeks post-treatment									
Infection intensity									
Light	16	18.0 (10.6–27.6)	25.5–943.5	0	─	0	1	1.1 (0.0–6.1)	25.5
Moderate	1	1.1 (0.0–6.1)	2,014.5	0	─	0	0	─	0
Heavy	0	0	0	0	─	0	0	─	0
Total positivity	17	19.1 (11.5–28.8)	25.5–2,014.5	0	─	0	1	1.1 (0.0–6.1)	25.5
Arithmetic mean EPG	140.5			0			0		
New positive individuals	0			0			1		
Cured individuals	31			12			13		
Cure rate (CR)	64.6			100			100		
Egg reduction rate (ERR)	94.2			100			100		
6 weeks post-treatment									
Infection intensity									
Light	22	24.7 (16.2–35.0)	25.5–918.0	0	─	0	0	─	0
Moderate	1	1.1 (0.0–6.1)	7,497	0	─	0	0	─	0
Heavy	0	─	0	0	─	0	0	─	0
Total positivity	23	25.8 (17.1–36.2)	25.5–7,497.0	0	─	0	0	─	0
Arithmetic mean EPG	250.2			0			0		
New positive individuals	2			0			0		
Egg-positive individuals after clearance of helminth eggs	7			0			0		

EPG = Eggs per gram

CI = Confidence interval

Arithmetic mean EPG, CR, ERR and re-infection rate do not include the new positive individuals at calculations

Classification of infection intensity of *T*. *trichiura*, *A*. *lumbricoides* and hookworm, respectively according to WHO cut-offs [39]

^¥^ 1–999 epg (light); 1,000–9,999 epg (moderate); ≥10,000 epg (heavy)

φ; 1–4,999 epg (light); 5,000–49,999 epg (moderate); ≥50,000 epg (heavy)

ᶲ 1–1,999 epg (light); 2,000–3,999 epg (moderate); ≥4,000 epg (heavy)

*T*. *trichiura* infections: At baseline, 48 out of 89 (53.9%; 95% CI = 43.0–64.6) were infected with *T*. *trichiura*. The infected individuals mainly suffered from light-to-moderate infection intensity (50.5%; 95% CI = 39.8–61.3) and 3.4% (n = 3; 95% CI = 0.7–9.5) had acquired heavy intensity of infection. At 3 weeks post treatment, the positivity rates of *T*. *trichiura* reduced from 53.9% (95% CI = 43.0–64.6) to 19.1% (95% CI = 11.5–28.8). The infections were mainly mild infections, and no heavy infection was observed. Of 48 infected individuals at baseline, 31 individuals were cured and the CR for *T*. *trichiura* infection was 64.6%. The egg reduction rate (ERR) was 94.2%. It is important to note that 7 participants of the 31 cured individuals showed increment of egg output at 6 weeks post treatment. Of the 7 individuals, one was a female and the rest were male and 3 were children aged below 12, while the other 4 were adults. Two new positive adult individuals were also identified at this time-point.

*A*. *lumbricoides* infections: Overall, 13.5% (n = 12; 95% CI = 7.2–22.4) of the participants were infected with *A*. *lumbricoides*. Most infections were of light-to-moderate intensity. Only one individual acquired heavy intensity of *A*. *lumbricoides* infection. At 3 weeks post-treatment, all the infected individuals were cured. The CR and ERR reached 100%. No infection was found at the second follow-up (6 weeks post-treatment).

Hookworm infections: At baseline, 14.6% (n = 13; 95% CI = 8.0–23.7) participants were positive for hookworm infections. Of 13 infected individuals, 9 had light intensity, one moderate intensity and 3 with a heavy intensity. At 3 weeks after treatment, all eggs were cleared (ERR = 100%), with a cure rate of 100%. A new positive individual was identified with a light intensity of hookworm infection at 3 weeks post-treatment but no infection was detected from this individual at 6 weeks post-treatment. No infection was observed at 6 weeks post-treatment.

### *T*. *trichiura* infection patterns

**Fig 3:**
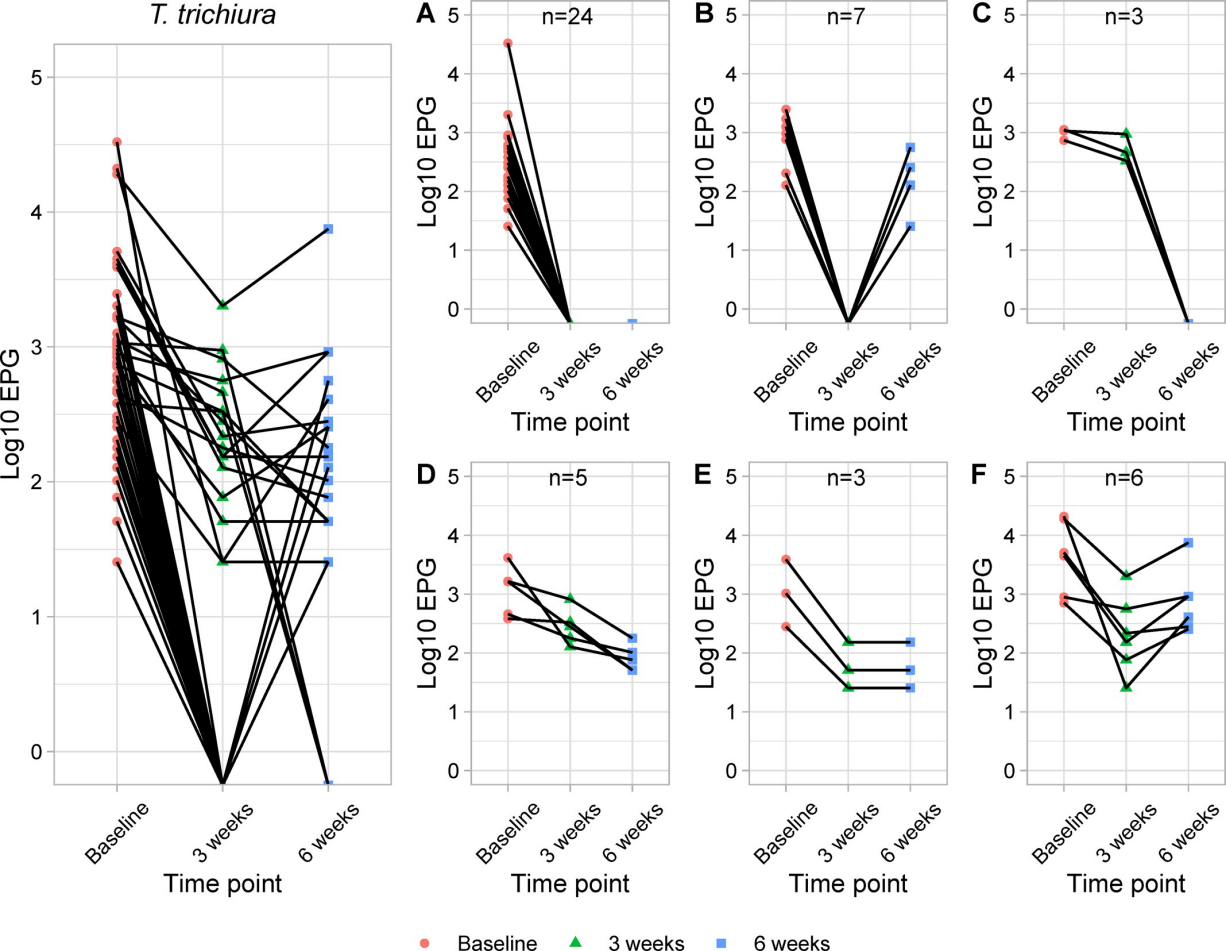
*T*. *trichiura* infection patterns over the three time points among *T*. *trichiura* positive participants at baseline (N = 48). Different patterns of *T*. *trichiura* infection were observed among 48 infected participants following 3 doses of albendazole treatment: (A) Overall *T*. *trichiura* infection patterns over 3 time points (n = 48). (B) cured from infections at 3 weeks and remain negative at 6 weeks (n = 24), (C) cured from infections at 3 weeks post-treatment but increased of egg output at 6 weeks post-treatment (n = 7), (D) egg output was reduced at 3 weeks post-treatment and become negative at 6 weeks post-treatment (n = 3), (E) showed reduction of egg output following treatment (n = 5), (F) showed reduction of egg output at 3 weeks post-treatment and remain unchanged at 6 weeks post-treatment (n = 3), (G) showed increment of egg output at 6 weeks post-treatment after a reduction at 3 weeks post-treatment (n = 6).

In the case of *T*. *trichiura* infections, different patterns of infection were observed among 48 infected individuals at baseline over the 3 time points (Fig 3A). The intensity of infection was found to be reduced or increased to different extents following the anthelmintic drug administrations and each infection patterns were shown in Fig 3B–3G. The proportion of each infection pattern were shown in S1 Table. At 3 weeks post treatment, all infected participants showed reduction of egg output and 31 of them (64.6%) were cured from *T*. *trichiura* infections (Table 5). As mentioned previously, 7 cured participants (14.6%) showed increment of egg output at 6 weeks post treatment (Table 5 and Fig 3C). Besides, 12.5% (n = 6) showed an increase of egg output after a reduction of egg output at first follow-up (Fig 3G), while 10.4% (n = 5) showed further reduction of egg output at 6 weeks post-treatment (Fig 3E). Surprisingly, there were 3 participants (6.4%) who were positive initially at first follow-up, however were egg negative at 6 weeks post-treatment (Fig 3D). Ascariasis and hookworm infection patterns were not described in the present study as all the infected individuals were cured after deworming treatment. No infection was identified at second follow-up.

## Discussion

The current study showed that STH infections are still a public health problem among indigenous Orang Asli communities in Malaysia. Prior to intervention, more than half (62.1%) of the participants were positive for at least one STH species (Table 2). Of the four surveyed villages, three villages were highly infected with STH (>50%). Nevertheless, an encouraging trend was observed in Bukit Bangkong village which was found to have the least infections (14.9%). From our observation, the houses in this village were mainly concrete house and equipped with toilet facilities. In contrast with the other three villages, some residents are living in traditional wooded house and lack of toilet facilities, especially Rasau and Sungai Judah villages. Additionally, all families from Rasau and Sungai Judah are found to have household income below the poverty threshold of Malaysia (RM2208). There were evidences that villages with less developed sanitation and high poverty rate had higher rate of infections.

Based on our findings, *T*. *trichiura* infections was found to be the most predominant among the three STH species. This observation was in agreement with previous studies conducted among Orang Asli communities in Malaysia [7–10, 40, 46–48]. Globally, *T*. *trichiura* predominance in Malaysia (49.9%) was reported in a systematic review [2]. Besides, *A*. *lumbricoides* has been reported in most previous studies as the second most common STH species infecting Orang Asli populations, followed by hookworm [7–10, 46–48]. However, the opposite trend was observed in the current study and two previous studies [36, 40] where the positivity rates of hookworm was higher than *A*. *lumbricoides*. The disparate outcome could be related to the nature of the soil in the study area which favours the development of infective stages of hookworm larvae, which lead to higher soil contamination with hookworm larvae [49–51]. However, further investigation on soil analysis is needed to confirm this postulation.

In the present study, it was also found that mixed infection is very common. This is not surprising, particularly in endemic areas due to the prolonged exposure to helminth infections. It needs to be highlighted the relatively higher infection rates of *T*. *trichuria* and hookworm (10.7%) coinfection as both species are transmitted via different routes of transmission (faecal-oral route and skin penetration, respectively). Additionally, about 5% of the study population harboured all three STH species at the time of the study. These findings were particularly crucial as individuals with multiple infections may increase the likelihood of suffering from multiple morbidity and at higher risk of significant helminth-related morbidity. Ezeamama et al. [52] and Robertson et al. [53] hypothesized that the concomitant of *Trichuris* and hookworm could attribute to higher burden of iron deficiency anaemia.

Poor sanitation has always been related to the prevalence of STH infections. A recent systematic review and meta-analysis study reported that improved sanitation was correlated with reduced prevalence of STH infections [54]. The current finding showed household latrine availability and indiscriminate defecation could be the causative factors. Open defecation near the streams or bushes is still commonly practiced among the communities even when possessing household latrine. Some were forced to practice open defecation when pursuing agriculture activities which is far away from home. With frequent habitual fecal contaminations, soil can be heavily contaminated with helminths eggs and/or cysts and serve as a reservoir for continued helminthiasis transmission and poses reinfection. Thus, good environmental sanitary and hygienic practices were important in preventing the spread of helminth infections.

In our study, it was noted that children and teenagers (<18 years old) were almost 4 times at risk of getting STH infections. Children and teenagers are at their age of being inquisitive and actively involved in outdoor activities, hence are consistently in contact with contaminants from their living environment, leading to higher exposure to sources of infection. Besides, children also lack the awareness of good personal hygiene practices and inadequate knowledge of consequences of exposure to parasitic organisms. Health education programmes on good personal hygiene practices as well as knowledge on STH transmission and preventions should be deliberated in school-based programme to instil good habits and curb the transmission at early age. Otherwise, the negative health consequences of the infection are likely to impact their wage-earning capacity or productivity if persist to adulthood.

Based on the intervention study, our results indicate that triple dose regimens showed moderate effect of curative rate against *T*. *trichiura* (CR 64.4%). Nevertheless, high ERR (94.2%) was achieved in current study. Although diverse triple dose CR against *T*. *trichiura* (ranged 19.6–83.0) has been reported in earlier studies from different geographical areas, consistent high ERR (88.0–97.4%) was attained with triple dose albendazole regimens [23, 25, 28, 55]. Given that CR against *T*. *trichiura* infections is relatively low, ERR should be taken into consideration in determining the drug efficacy as it is the indicator that determines infection intensity which reflects the reduction of morbidity.

Unlike *T*. *trichiura*, past studies showed single dose of albendazole is enough to treat *A*. *lumbricoides* infections [21, 56]. As expected, our finding revealed a satisfactory result against *A*. *lumbricoides* infection with triple dose of albendazole treatment, where the CR and ERR were both found to be 100%, respectively at 3 weeks post-treatment. Other studies conducted in China, Indonesia and Laos also reported high CR, ranging from 91% to 99% and ERR ranging from 88% to 100%, respectively [25, 55, 57, 58]. With regards to hookworm infections, 100% for both CR and ERR were attained with triple dose albendazole. Similarly, Sungkar et al. [58] also reported 100% for both CR and ERR by triple dose albendazole treatment. Likewise, high curative rate and ERR (>90%) against hookworm has been reported in one local study and other countries [23, 25, 55, 59].

Based on previous comparative studies, triple dose regimen possessed considerably higher curative effect against STH than single or two doses regimen [23, 25], suggesting the necessity for triple dose to achieve adequate curative rate against STH. A study in China reported higher CR with triple dose regimen of albendazole compared to single dose against *T*. *trichiura* (33.8% vs 56.2% for one versus three doses) and hookworm (69.1% vs 92%). Another study in Gabon attained an even higher CR (83%) with triple dose albendazole when compared with single dose (CR 40%) against *T*. *trichiura* while a minimum of two dosage were required to achieve satisfactory performance against hookworm infection [23]. Nevertheless, single dose regimen was enough to treat *A*. *lumbricoides* infection [23, 25]. Hence, in area with high co-endemicity of the three STH species, triple dose regimen of albendazole is desired over single dose regimen.

A particular observation at 6 weeks post treatment was the rapid increment of *T*. *trichiura* egg output after clearance of *T*. *trichiura* eggs at 3 weeks post treatment. One possible explanation for this treatment failure could be the incomplete action of ovicidal, larvicidal or vermicidal effect of albendazole against *T*. *trichiura*. The ability of *T*. *trichiura* to embed itself into the intestinal mucosa may prevent them from being eradicated despite being paralysed by albendazole drug. This allows them to avoid the action of drug and recover after the treatment as *T*. *trichiura* is thought to be able to excrete the drug through P-glycoprotein-mediated transport [60].

It is possible that repeated treatment with albendazole may alter reproductive activity and promote anthelmintic resistance. Traits such as parasite survival and fecundity may evolve in response to selection, with trade-offs between traits limiting their evolution [61]. Under the selection pressure of anthelmintic drugs, natural selection may favor worms that mature earlier to produce eggs [62], which could shorten their life cycle but result in smaller worms and reduced fecundity [63].

Besides, a recent study on *Gnathostoma spinigerum* reported the survival of immature pre-adult worm in human following albendazole treatment [64]. In that study, alteration on integument surfaces was noticed from surviving albendazole-treated larvae, postulated structural adaptation induced by the worm to protect against effect of albendazole. Similar phenomenon might occur in *T*. *trichiura* species resulting in the survival of drug-treated larvae. However, this needs to be further confirmed by studies on morphological changes in drug-treated larvae of *T*. *trichiura*.

On the other hand, it is unclear why 3 individuals who were initially egg positive became egg negative at 6 weeks. The possible explanation could be the low sensitivity of diagnostic technique. Despite being a gold standard of diagnostic method, Kato-Katz is known to have low sensitivity against light intensity helminth infections. These individuals might have been misdiagnosed due to low number of eggs in the light intensity infection following treatment, especially with single fecal smear examination [65]. Intermittent shedding of eggs by helminth and non-equal distribution of helminth eggs in stool samples further lead to low sensitivity of stool examination [66, 67]. Meanwhile, the new positive individuals may either be positive from new infections or from a false negative result with the previous time-point because of daily variations in egg output and the lower sensitivity of the Kato-Katz assay.

In line with the main objective of preventive chemotherapy to reduce the morbidity of STH infection, 3-day regimen of albendazole possess higher ERR against *T*. *trichiura* is more ideal. Yet, triple dose is deemed not feasible for mass administration owing to higher cost, challenges on compliance rate and logistic issues, particularly in remote area. Hence, the employed drug regimen needs to adopt current prevailing conditions to strike a good balance between feasibility and improvement of treatment efficacy. For population with high intensity of *T*. *trichiura* infections, triple dose is desired, at least at the initial stage of treatment. Otherwise, single dose is sufficed. Targeted and customised treatment regimen is crucial as one size fits all regimen is not sustainable and effective. Moreover, a proper database and monitoring system pertaining to anthelmintic drug treatment is suggested for the ease of monitoring and adjustment of drug dosage and frequency of drug administration based on current prevalence rate and morbidity in endemic population. Meanwhile, it is recommended to further investigate alternative ways, such as combination therapy for better curative effect of *T*. *trichiura*.

We acknowledge some limitations of the current study. Firstly, the sample size of this longitudinal study was relatively small for an intervention study due to the requirement of paired samples for the 3 time points. However, planned efforts to increase the sample size in 2020 were hampered by the Covid-19 pandemic. This could reduce the power of study and affect the reliability of outcomes. Besides, single slide reading could affect the detection rate of helminthiasis due to the low sensitivity of Kato-Katz techniques, however, we have tried to increase consistency by having each slide examined by 2 personnel.

## Conclusion

This study reaffirms that STH infections are still highly prevalent among Orang Asli population, although a relatively low infection rate was identified in one study village. Despite mass deworming program being emphasised as the main strategy in controlling STH, results may be more promising when it is combined with integrated approaches to consolidate the gains of deworming program. Integrated control strategies should focus on improvement of environmental sanitation, particularly on access to sanitation facilities and community-based WASH (water, sanitation and hygiene) intervention. Additionally, health education on important of sanitation and good personal hygiene should specifically focus on school-based programme. In the context of mass deworming programme, it is paramount for treatment regime whether single dose or multiple doses to be targeted and customised to specific areas or villages in order to optimise resources. For substantial reduction of *T*. *trichiura* burden in populations with very high burden, triple dose albendazole is recommended. The rapid increase of *T*. *trichiura* egg output at 6 weeks further add to the challenge of reducing the burden of this neglected disease. Meanwhile, further investigation on alternative ways, such as combination therapy or effective drugs for better management of *T*. *trichiura* should be considered.

## Supporting information

S1 Checklist Questionnaire of inclusivity in global research. (DOCX)

S1 Table Proportion of *T*. *trichiura* infection patterns after deworming treatment among *T*. *trichiura* infected participants at baseline (N = 48). (DOCX)

S1 Appendix Baseline STH epidemiology dataset. (XLSX)

S2 Appendix Dataset on prevalence and intensity of STH infections over 3 time-points. (XLSX)
