# Supplementary material for: Efficacy of triple dose albendazole treatment for soil-transmitted helminth infections
Source: PLoS One. 2022 Aug 12;17(8):e0272821. doi: 10.1371/journal.pone.0272821 (PMC9374461; doi:10.1371/journal.pone.0272821)
Supplement: S1 Table — (DOCX) [file pone.0272821.s002.docx]

**S1 Table. Proportion of *T. trichiura* infection patterns after deworming treatment among *T. trichiura* infected participants at baseline (N=48)**

| **Patterns of infection** | **n (%)** | **95% CI** |
| --- | --- | --- |
| Cured at 3 weeks and remained negative at 6 weeks | 24 (50.0) | 35.2-64.8 |
| Cured at 3 weeks but increased intensity at 6 weeks | 7 (14.6) | 6.1-27.8 |
| Reduced intensity at 3 weeks but increased intensity at 6 weeks | 6 (12.5) | 4.7-25.3 |
| Reduced intensity at 3 weeks and 6 weeks | 5 (10.4) | 3.4-22.7 |
| Reduced intensity at 3 weeks and become negative at 6 weeks | 3 (6.3) | 1.3-17.2 |
| Reduced intensity at 3 weeks and intensity remains unchanged | 3 (6.3) | 1.3-17.2 |
